# VirusTAP: Viral Genome-Targeted Assembly Pipeline

**DOI:** 10.3389/fmicb.2016.00032

**Published:** 2016-02-02

**Authors:** Akifumi Yamashita, Tsuyoshi Sekizuka, Makoto Kuroda

**Affiliations:** Pathogen Genomics Center, National Institute of Infectious DiseasesTokyo, Japan

**Keywords:** NGS, viral genome, *de novo* assembly, web service, host genome subtraction

## Abstract

Although next-generation sequencing (NGS) technology provides a comprehensive means with which to identify potential pathogens from clinical specimens, simple and user-friendly bioinformatics pipelines are expected to obtain the entire viral genome sequence, subsequently providing traceability, based on extensive molecular phylogenetic analyses. We have developed a web-based integrated NGS analysis tool for the viral genome (virus genome-targeted assembly pipeline: VirusTAP), which includes extensive sequence subtraction of host- or bacteria-related NGS reads prior to *de novo* assembly, leading to the prompt and accurate assembly of viral genome sequences from metagenomic NGS reads. The VirusTAP web site is at https://gph.niid.go.jp/cgi-bin/virustap/index.cgi/.

## Introduction

As next-generation sequencing (NGS) technology is becoming a more common means with which to detect pathogens from patients or clinical samples, the need for simple and effective bioinformatics pipelines for the analysis of NGS reads has increased in parallel. One of the major difficulties in this process is the correct *de novo* assembly of viral genomes from crude metagenomic deep sequencing reads, including large amounts of bacteria and human related sequencing reads. Such read contaminations often force the server to overload during *de novo* assembly and might cause mis-assembly of the resultant contigs. Pre-filtering by host-mapping subtraction could lead to efficient *de novo* assembly, allowing the rapid and accurate procurement of a complete viral genome sequence. In addition to the accuracy of *de novo* assembly, the exclusion of human-related sequences can circumvent conflicting ethical issues by avoiding analyzing the personal genetic information of patients.

To facilitate the steps that require computational resources and skill, we constructed a web-based integrated NGS analysis tool for the viral genome (virus genome-targeted assembly pipeline: VirusTAP) by performing the following informatics steps: (1) quality trimming and adaptor removal, (2) read subtraction of host- and bacteria-related sequences by a read-mapping method, and (3) *de novo* assembly using multiple combinations of recently advanced assemblers. On the VirusTAP website, users can complete viral genome assemblies from raw NGS reads just by clicking several selections. VirusTAP is freely available for academic users from the following website: https://gph.niid.go.jp/cgi-bin/virustap/index.cgi.

## Materials and Methods

### Clinical Specimens and NGS Short Reads for Viral Genome Assembly

Sample NGS short reads were prepared using stool specimens from a patient with rotavirus gastroenteritis and analyzed as described previously (Mizukoshi et al., 2014). In brief, total RNA was prepared from patient feces, followed by RNA-seq library preparation using a ScriptSeq v2 RNA-seq library preparation kit (Epicentre, Madison, WI, USA). Deep sequencing was performed to obtain 120-mer paired-end (PE) short reads with a MiSeq Reagent kit v2 (Illumina, San Diego, CA, USA). The sample short-read sequences have been deposited in the DNA Data Bank of Japan (DDBJ; accession number: DRA004165).

The study protocol was approved by the institutional medical ethics committee of the National Institute of Infectious Diseases in Japan (Approval No. 576), and it was conducted according to the Declaration of Helsinki Principles.

### NGS Read Processing

**FIGURE 1:**
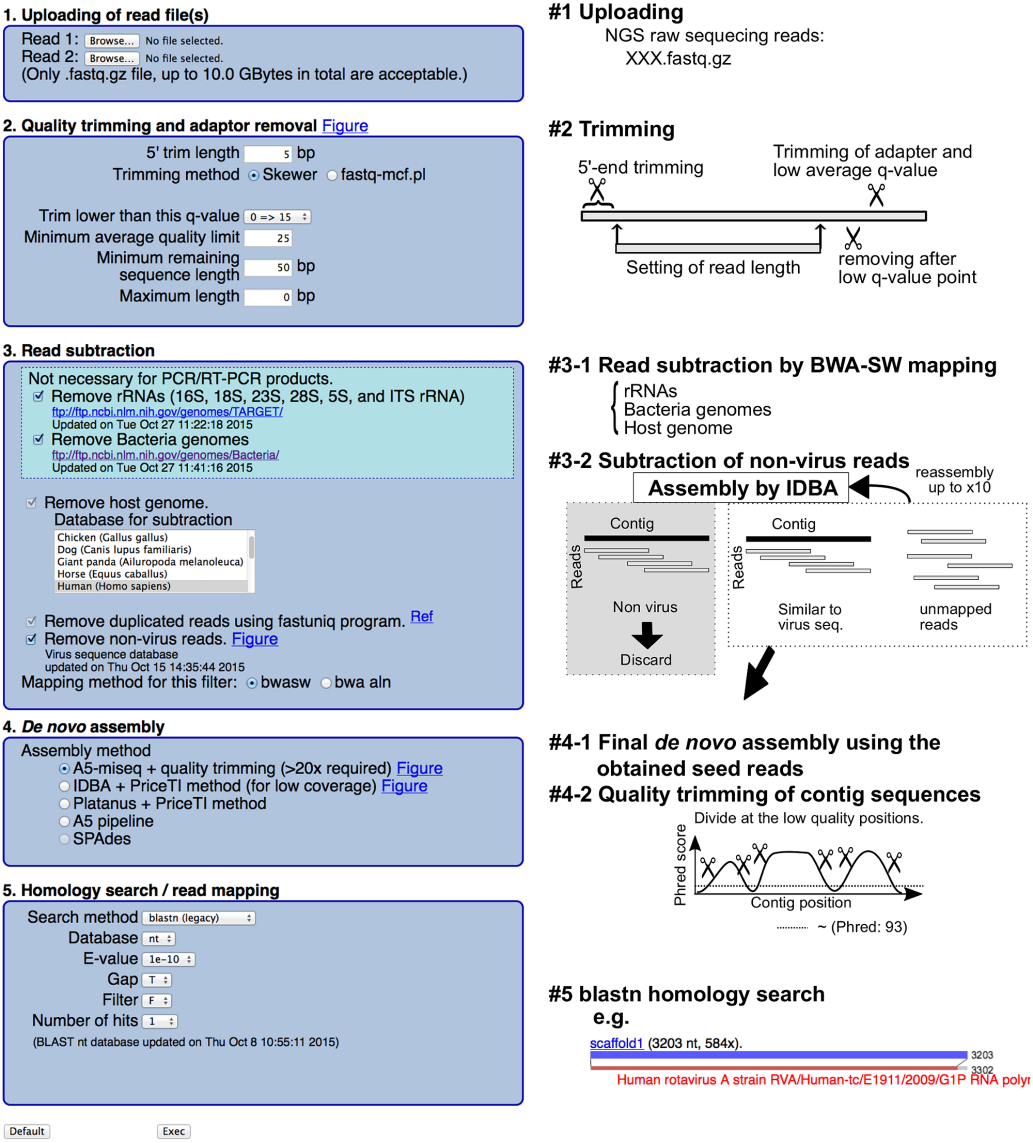
**Schematic representation of the VirusTAP procedures**.

VirusTAP accepts single- and PE reads (#1 in **Figure 1**). Read quality trimming was performed using the skewer (Jiang et al., 2014) or fastq-mcf program in the ea-utils package^1^, with an additional trimming filter for unreliable sequences after a user specified quality score (#2 in **Figure 1**). Host read subtraction by read-mapping was performed with the bwasw program (version 0.7.9a-r786; Li and Durbin, 2009) against ribosomal RNAs (16, 18, 23, 28, 5S and internal transcribed spacers rRNA were retrieved from the following ftp site^2^), bacterial genome sequences^3^ and the latest host organism genome sequences^4^ (#3–1 in **Figure 1**).

To further remove residual non-virus sequence reads, the above subtracted PE reads are assembled with IDBA-UD (Peng et al., 2012), followed by the filtering of non-virus PE reads with megablast (v. 2.2.26) (Altschul et al., 1997) and RAPSearch2 (v. 2.16) (Zhao et al., 2012) search against the customized viral nucleotide/protein sequences from the NCBI nt/nr database based on virus taxonomy, excluding bacteriophages^5^ (#3–2 in **Figure 1**). No significant *e*-value less than either 1*e* – 10 for megablast or *e* – 30 for RAPSearch2 was determined for non-virus contigs, and residual PE reads were further subtracted for the following process. This non-virus filtering step will be repeated up to 10 times or until non-virus contigs disappear. After removing the non-virus sequencing reads, broken PE reads are also removed for the following *de novo* assembly.

The *de novo* assembly pipeline can be selected from the following four pipelines (#4–1 in **Figure 1**): A5-miseq (Coil et al., 2015), Platanus (version 1.2.1) (Kajitani et al., 2014) with PriceTI (Ruby et al., 2013), and IDBA-UD (Peng et al., 2012) with PriceTI (Ruby et al., 2013). PriceTI (v. 1.2) (Ruby et al., 2013) performs PE iterative contig extension from pre-assembly contigs by Platanus or IDBA-UD.

A5-miseq is one of the most recommended *de novo* assemblers for general NGS PE reads because assemble accuracy for the resultant scaffolds can be checked using the resulting “XXX.scaffolds.fastq” file based on the read-mapping score. The contigs were divided at the nucleotide position, where the Phred score is less than 93 and the resulting sequences shorter than 100 bp are removed. The final scaffolds were subjected to bwasw read mapping and a megablast homology search against the NCBI nt database; the alignment results are visualized at the web page.

## Results and Discussion

### Basic VirusTAP Procedure

Briefly, a schematic representation of VirusTAP is shown in **Figure 1**. The VirusTAP process starts with the quality trimming, followed by host- and bacteria-related read subtraction. The read subtraction is performed in the following two steps: first, read-mapping with the bwasw program to rRNA sequences, bacterial genome sequences, and selected virus-host genomes, such as human, mouse, or monkey, was performed; second, non-virus filtering against a virus nucleotide/protein database, excluding bacteriophages, can be selected for further subtraction to extract possible virus-related NGS reads. The above-described highly extensive non-virus read subtraction facilitates effective *de novo* assembly and prompt analysis, achieving both time savings and accuracy.

### VirusTAP Performance Comparison with Direct Metagenomic Assembly Methods

**FIGURE 2:**
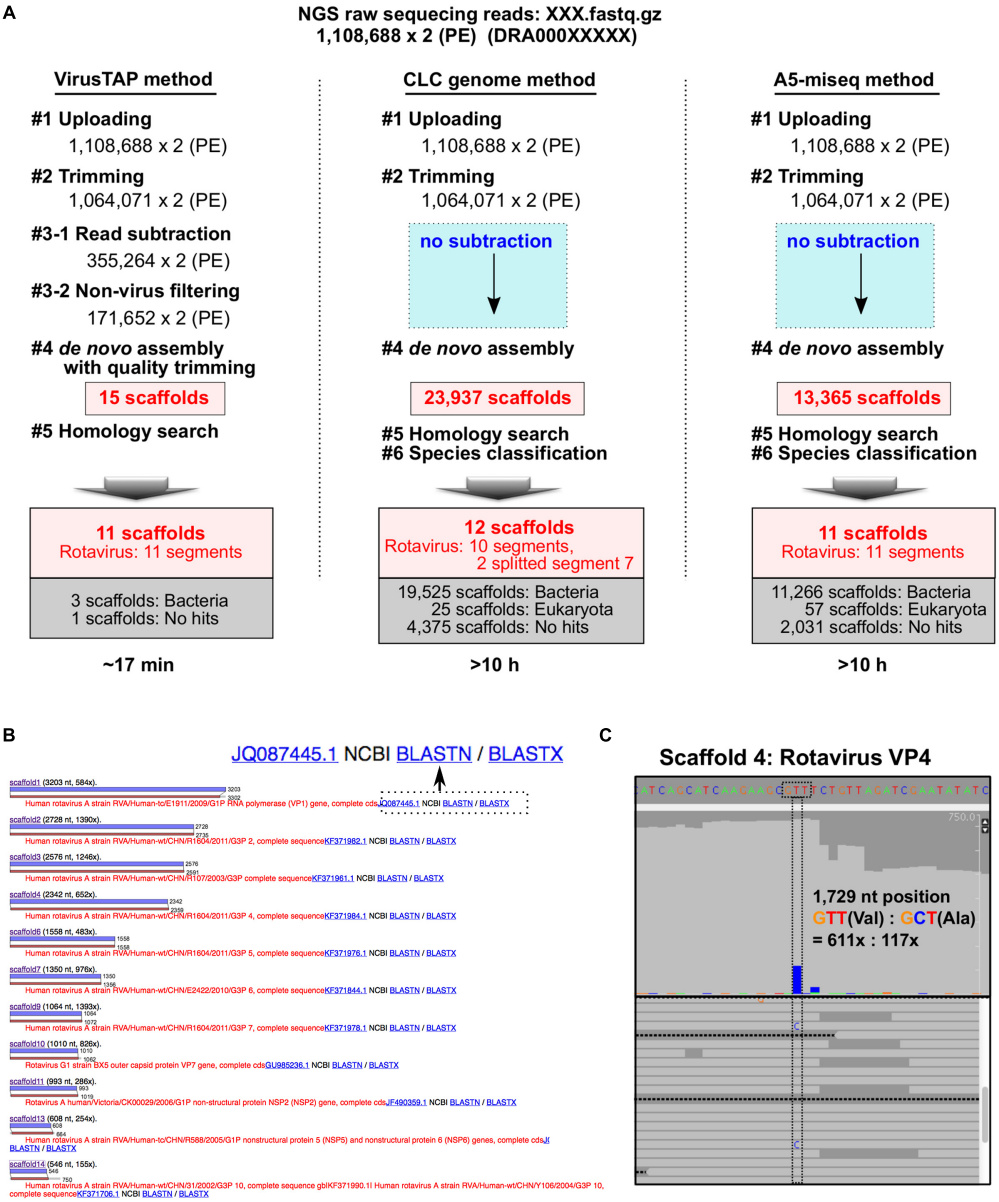
**Performance comparison of VirusTAP with non-subtraction methods. (A)** Sample metagenomic RNA-seq reads obtained from the rotavirus gastroenteritis patient were analyzed by VirusTAP and non-subtraction methods. **(B)** Pair-wise alignment view of the most similar hit for each scaffold. Eleven scaffolds showed similarity to eleven segments of rotavirus genome sequences. NCBI Web blast search (BLASTN or BLASTX) can be directly performed for each scaffold by clicking the button to reconfirm the similarity. **(C)** Visualization of the read-mapping result in the scaffold 4. Viral quasispecies and read coverage on each scaffold can be confirmed by the read-mapping viewer using the bam file in the downloaded results package.

Using metagenomic RNA-seq short reads obtained from stool specimens from a patient with rotavirus gastroenteritis (Mizukoshi et al., 2014), the performance of VirusTAP was compared with that of other *de novo* assembly methods without subtraction treatment. For instance, direct metagenomic assembly without subtraction using a CLC genome workbench v.8.5.1 (QIAGEN, Aarhus Denmark) or A5-miseq produced 23,937 or 13,365 scaffolds, respectively; VirusTAP significantly reduced the results to 15 scaffolds (**Figure 2A** step 4).

Those direct metagenomic assembly methods generated unexpectedly abundant scaffolds related to multiple organisms at step 4 in **Figure 2A**. In particular, over 80% of scaffolds were composed of bacterial sequences (19,525 and 11,266 scaffolds by CLC genome and A5-miseq, respectively; **Figure 2A**), suggesting that subsequent homology searches and taxonomy classifications are very laborious and time-consuming procedures to identify the final viral genome sequence. Indeed, the entire VirusTAP process for these sample reads required ∼17 min to obtain 11 segments of the rotavirus genome sequence, whereas the other two methods required more than 10 h, because of the time-consuming *de novo* assembly and homology search for all abundant scaffolds (**Figure 2A**).

In addition to the stool sample, human serum specimen of Dengue fever patient was investigated by RNA-seq, followed by VirusTAP analysis. Since bacterial contamination is not common in serum, plasma, spinal fluid, and urine specimens, VirusTAP efficiently removed the more than 90% of host-related sequence in total reads to obtain possible virus sequence reads (data not shown). Only human genome subtraction could be sufficient for the serum specimen, but fecal or pharyngeal specimens contain unfavorable bacterial reads for virus genome assembly. This study demonstrated that rather laborious sample such as bacteria rich stool sample can be acceptable to determine a virus genome by VirusTAP, it includes non-virus filtering (see step #3–2 in **Figure 1**) to facilitate the virus genome assembly.

The results of the homology search of pair-wise alignment for each scaffold can be visualized on the web (**Figure 2B**) or by downloading the full-results package, although VirusTAP provides the results against the latest NCBI nt database. Thus, users can reconfirm the obtained scaffolds by NCBI BLASTN or BLASTX web search with a direct web link (**Figure 2B**). The downloaded results package includes the bwa-mapping “sorted.bam” file, which can be imported into the reads-mapping viewer to visualize nucleotide polymorphisms, insertion-deletion and read coverage depth. In this sample study, a T/C heterogeneous mixture was identified at the 1,729 nt position of scaffold 4 (**Figure 2C**), indicating a possible quasispecies with a valine or alanine residue at the 578 aa position of the VP4 protein. VP4 is a spike protein at the outer capsid and has receptor binding activities and functions in membrane penetration (Trask et al., 2012), suggesting that the quasispecies could emerge during rotavirus gastroenteritis, contributing to the antigenic drift during rotavirus infection.

## Author Contributions

TS and MK performed the metagenomic sequencing and statistical and bioinformatics analyses. AY and MK participated in the design of the study, performed the statistical analysis, and drafted the manuscript. All authors read and approved the final manuscript.

## Conflict of Interest Statement

The authors declare that the research was conducted in the absence of any commercial or financial relationships that could be construed as a potential conflict of interest.

## Footnotes

1 http://code.google.com/p/ea-utils/

2 ftp://ftp.ncbi.nlm.nih.gov/genomes/TARGET/

3 ftp://ftp.ncbi.nlm.nih.gov/genomes/all/

4 ftp://ftp.ncbi.nlm.nih.gov/genomes/

5 ftp://ftp.ncbi.nih.gov/pub/taxonomy/
